# Knowledge of Emergency Contraception and Contraceptive Practices: Representative Study from Rural Uttar Pradesh

**DOI:** 10.4103/0970-0218.69293

**Published:** 2010-07

**Authors:** Aruna Nigam, Neha Maheshwari, Anupam Prakash

**Affiliations:** Department of Obstetrics and Gynaecology, Lady Hardinge Medical College and Associated Hospitals, New Delhi, India; 1 Intern, Era’s Lucknow Medical College and Hospital, Lucknow – 226 003, Uttar Pradesh, India; 2 Department of Medicine, Lady Hardinge Medical College and Associated Hospitals, New Delhi, India. E-mail: prakashanupam@hotmail.com

Sir,

Unprotected sexual intercourse and contraceptive method failure lead to unintended pregnancies. Emergency contraception (EC) holds promise in such situations. This study aimed to assess the awareness and level of knowledge toward EC among 300 married women in the reproductive age group (18–45 years of age) seeking routine gynecological or obstetric care at Era’s Lucknow Medical College and Hospital, Lucknow. A prospective cross-sectional study was conducted employing an instructor-administered pretested prestructured questionnaire after a written informed consent. The questionnaire included information about the age and education of husband and wife, obstetric profile, knowledge and use of contraceptives, and awareness and use of EC.

Table 1 outlines population characteristics. Family size of the respondents had an inverse association with both the wife’s education and husband’s education (*r* = −0.328, *P*<0.001 and *r* = −0.123, *P*=0.034, respectively). Abortion was sought by 23% of the respondents at some time or the other.

**Table 1:** Demographic characteristics of the study group (*n*=300)

Population features	Frequency (%)
Age (years)	
<20	26 (8.66)
21–30	216 (72)
>30	58 (19.33)
Education – wife	
Uneducated	102 (34)
Primary	38 (12.66)
Matriculation and senior secondary	124 (41.33)
Graduate and postgraduate	36 (12)
Education – husband	
Uneducated	66 (22)
Primary	14 (4.66)
Matriculation and senior secondary	154 (51.33)
Graduate and postgraduate	44 (14.66)
Number of living children	
None	92 (30.66)
1	90 (30)
2	48 (16)
3	38 (12.66)
More than 3	32 (10.66)
History of voluntary termination of pregnancy	
Once	56 (18.6)
Twice	8 (2.6)
Three times	8 (2.6)

The two-children norm was supported by 38%; 45% believed in 3, 9.3% believed in more than three children, while 7.3% were non-committal. None supported the view of one-child norm. Ideal family size as opined by the responders was directly related to their educational status (*r* between “ideal family size” and “wife’s education” = −0.285, *P*<0.001). In fact, the women who had borne more children believed in a larger family size (*r* between “parity” and “ideal family size” = 0.245, *P*<0.001).

Table 2 shows the contraceptive trends in the study population. Condom as a method of contraception was known to most (87%), although, its usage was barely 24%. Only 36% were regularly using some method of contraception. Remarkably, three quarters of females were aware of all the methods of contraception, but there was a wide gap between awareness and practice.

**Table 2:** Contraceptive practices in the study group (*n*=300)

	Knowledge (%)	Practice (%)
Condom	262 (87.3)	74 (24.6)
IUD	242 (80.7)	14 (4.6)
Oral contraceptives	244 (81.3)	24 (8)
Vasectomy	220 (73.3)	Nil
Tubectomy	228 (76)	Nil

Only six women (2%) were aware of EC pills and two of them had used it. Once the respondents were aware of the availability of this method, all the women expressed their willingness to use the EC pills in future, if the need arose.

The present study is an eye opener; despite 65% literacy among females, only 2% were aware of EC. Hence, EC is an area which needs to be publicized. In Chandigarh, of women seeking abortion, only 1% knew of EC(1) and in a New Delhi study, none was aware of EC.(2)

According to NFHS-III, knowledge about various temporary and permanent methods among men and women ranges from 45% to 97%, which corroborates with the findings of this study, with the knowledge ranging from 73% to 87% about various methods. According to NFHS III, the knowledge about EC is 20% in men and 11% in women. This study reflects much lower knowledge among women (2%), and this may be attributed to the study being conducted in primarily a rural population, that too in one of the BIMARU states. There has to be a greater emphasis and motivation to increase the drive regarding the contraception.

There is enormous potential for EC in reproductive health of the country. The community health worker can play an important role by percolating the knowledge of EC deep down in the community. Workers can act as a depot holder for EC as they do for oral contraceptives and condoms. Emergency contraception gives a woman the last opportunity to protect herself from an otherwise unprotected intercourse; it has the potential to achieve the goal of “all pregnancies should be wanted” and can be a handy tool to achieve the objectives of National Population Policy.
